# Editorial: Sport governance: challenges, developments and future pathways

**DOI:** 10.3389/fspor.2026.1998764

**Published:** 2026-10-05

**Authors:** Roy McCree, Umer Hussain, Veena Mani

**Affiliations:** 1 The University of the West Indies St. Augustine, St. Augustine, Trinidad and Tobago; 2 Missouri State University, Springfield, MO, United States; 3 UL Research Kozhikode, India, Kozhikode, India

**Keywords:** equality, equity, inclusion, intellectual property, representation, sport for development, sport governance

Editorial on the Research Topic Sport governance: challenges, developments and future pathways

## Introduction

Emerging challenges, including corruption scandals in sport and debates surrounding gender transition, have renewed attention on the importance of effective sport governance—the central theme of this special issue of *Frontiers in Sports and Active Living*. The issue consists of 13 papers that deal with a wide range of important topics across several countries, including Australia, the USA, Germany, China, Korea, Tunisia and Portugal. Although the various articles may overlap or intertwine, given the broad scope of sport governance, they can possibly be classified or categorized around six major themes based on their particular focus. These include: state involvement in sport, the Olympics, athlete development, corporate Governance, intellectual property rights and sport for development.

The state-oriented articles deal with the issues of public subsidies for professional soccer in Germany, sport policy development in Australia, state intervention in US College Sports and local government in China and Portugal. In their study entitled “Public Subsidies for Professional Football Clubs: A Conceptual Political Economy Analysis,” Baumeister et al. provided a more integrated conceptual framework to better understand the critical importance of state subsidies in professional football based on a study of Germany's Bundesliga. In making a case for such public subsidies, they strongly suggest that they should not be narrowly conceived as just a strategy to rescue clubs in crisis but as part of a broader sustainable developmental strategy or catalyst to promote urban development, economic growth, social cohesion, and community identity or identity formation as a whole (1).

With respect to the USA, Hawkins interrogates the socio-political significance or fallout of one of Donald Trump's many Executive Orders (EO), in his article entitled, “Donald Trump's Name, Image, and Likeness (NIL) Executive Order: A Neo-Jacksonianism Tradition.” While the EO was seen as impacting all athletes in the major revenue-generating sports of American football and basketball, it was seen as having a disproportionate impact on black students who dominate participation in these two sports, which serves to reinforce the broader racialized status quo. Theoretically, the paper makes an important contribution to the study of populism in sport and populism in general and methodologically, to the use of qualitative research in sport through the application of contextual analysis. In the paper, “Australian Sport Policy and Advocacy: An Historical Account of Policy Evolution, Government Involvement, and the Advent of Lobbying,” Mountifield examines the Australian state's transition from a limited role in sport to a more active and interventionist one through the creation of formal sport policies, government sport bureaucracies, ministries and ministers responsible for sport, and dedicated funding for both elite and mass sport. The paper makes an important contribution to the literature on sport policy as well as the role of advocacy or lobbying in the policy process.

In the context of China, Li, Zhou, and Liu examined not simply the state, but the dynamics between the Central and Local Government in their article, “Top-down pressure and bottom-up responses: a study on provincial governments’ adoption and textual reproduction of sports industry policies in China.” The authors highlighted that policy implementation is not simply a matter of adopting national directives; rather, provincial governments interpret, modify, or closely reproduce policies according to institutional pressures, resource capacity, local development priorities, and adaptation needs. The article contributes by assessing how central sports policies are translated into action at the local government level. Outside of China, Sesinando, Seguí-Urbaneja and Teixeira analyzed municipal sport governance in Portugal in their article, “Local governance of sport: a national survey on how public opinion evaluates municipal sports development and priorities.” The study shows broad agreement between genders regarding satisfaction with municipal sports development, services, and facilities but finds weaker results on transparency. The study thus calls for greater transparency as well as improved public communication, and more systematic evaluation of municipal sport policies.

Three of the papers examined dealt with the Olympic Games as they relate particularly to the IOC, Olympic values and the impact of Olympic hosting on athlete performance. In their unprecedented historical examination of “The evolution of IOC membership and governance” from 1894 to 2023, Xu and Shi show how both internal and external factors of an economic, political, ideological, social and organizational nature combined to bring about substantive changes in the composition and operations of the IOC. As a consequence, the IOC has become much less Eurocentric, more meritocratic and representative, in relation to the presence of women, developing nations and athletes, although the authors question whether some of these changes, particularly the involvement of athletes, are more *symbolic* or cosmetic than real**.** In the article, “*Beyond excellence, friendship and respect: Olympic education stakeholders’ priorities for values in a changing world*,” Theodorakis et al. examine how Olympic education stakeholders recognise and seek to further contemporary values including peace, social inclusion, environmental sustainability, and gender equality, in addition to the traditional Olympic values of excellence, friendship, and respect. This study is highly relevant to sports governance as it helps to see how shifts in society shape attitudes within the major sports movement, similar to the paper by Xu and Shi.

In their paper entitled “Beyond the Podium: The Long-Term Impact of Hosting the Olympic Games on National Sporting Performance,” Schoettl and Kainz shift from issues of values and representation to the relationship between hosting and medal outcomes. In this regard, they argue that Olympic hosting should be understood not merely as a short-term home advantage but as a strategic instrument of sports policy. The study contributes to the literature by showing that Olympic hosting can work as a policy lever whose benefits are shaped by governance capacity, strategic prioritization, and sustained investment rather than by the event itself. The article by Guidotti et al. entitled “*Sustainability of top-performance athletes’ mental health and career retirement support services: European major sports organizations’ perspective*” examines much broader and important issues related to the (non)provision of formal support mechanisms, both financially and emotionally, to athletes in order to help them deal with life after sports. They argue that the available services in Europe are directed primarily towards the medal winners, which leaves a significant number of athletes outside the purview of support. This study signals an emerging trend of recognition for mental health support for elite athletes.

Kwon's paper entitled “*Athlete Data Sovereignty*” examines the equally important issue of who owns and controls the performance data produced by athletes during training or competition (e.g., heart rate output, reaction times). Reminiscent of Marx's notion of the alienation of workers from their own labour, it is argued that since such data are an extension of the athletes' identity and not some impersonal external output, they should be allowed to exercise greater *sovereignty* over them. The paper assumes great relevance in the age of AI and complements the issue of athlete representation raised by Xu and Chi in their paper on the IOC. In their paper entitled “*Whose game is it anyway? Palworld and the new frontier of intellectual property in eSports*,” Vargas-Chaves et al. also deal with the issue of property rights but in the particular context of e-sports or digital sports. Through the Palworld case, especially its perceived similarities with Pokémon, the article examined the tension between creative inspiration and infringement, while distinguishing between copyright, patent protection, trademarks, and Trade Dress. This article elucidates that legal regulation, platform governance, and intellectual property management are now central to the sustainability and integrity of the eSports industry.

In the only paper that focuses on national sport organizations, Ghodhbani and Souissi explore different governance models in the context of non-profit sports Federations in Tunisia in their article, “*Governance of Tunisian sports organizations: what is the matter?*” They argue that these Federations work within the oversight of both supranational and national regulatory systems that allow for both possibilities and constrictions. Majorly, they show how different governance models such as the *strong presidential model*, the *couple's presidential model*, the *exploded presidential model*, and the *managerial presidential model* influence the functioning of the Federations.

The paper by Westerbeek et al. can be situated more in the context of sport for development due to its focus on “*The costs of participation in and delivery of community sport in Australia.*” Based on a rigorous scoping or narrative review, the authors identify the economic and non-economic barriers to participation in community sport in Australia, critically examine existing strategies to deal with them and make several important policy recommendations to facilitate more participation. The paper should make an important contribution to the literature on sport policy as well as the growing field of sport for development.

The publication of this issue on Sport Governance comes at a time when FIFA is again under scrutiny due to several issues related to the hosting of the recently concluded World Cup of Soccer as well as the subsequent controversial investment plan, since shelved, to further commercialize the “beautiful game.” The controversies have served to further highlight the importance of “good” Governance in sport and the need for continuous research or investigation into its decision-making processes across both private, public, and NGO sectors.
